# Shifts in the Microbial Community Composition of Gulf Coast Beaches Following Beach Oiling

**DOI:** 10.1371/journal.pone.0074265

**Published:** 2013-09-10

**Authors:** Ryan J. Newton, Susan M. Huse, Hilary G. Morrison, Colin S. Peake, Mitchell L. Sogin, Sandra L. McLellan

**Affiliations:** 1 School of Freshwater Sciences, Great Lakes WATER Institute, University of Wisconsin-Milwaukee, Milwaukee, Wisconsin, United States of America; 2 Josephine Bay Paul Center, Marine Biological Laboratory, Woods Hole, Massachusetts, United States of America; Argonne National Laboratory, United States of America

## Abstract

Microorganisms associated with coastal sands serve as a natural biofilter, providing essential nutrient recycling in nearshore environments and acting to maintain coastal ecosystem health. Anthropogenic stressors often impact these ecosystems, but little is known about whether these disturbances can be identified through microbial community change. The blowout of the Macondo Prospect reservoir on April 20, 2010, which released oil hydrocarbons into the Gulf of Mexico, presented an opportunity to examine whether microbial community composition might provide a sensitive measure of ecosystem disturbance. Samples were collected on four occasions, beginning in mid-June, during initial beach oiling, until mid-November from surface sand and surf zone waters at seven beaches stretching from Bay St. Louis, MS to St. George Island, FL USA. Oil hydrocarbon measurements and NOAA shoreline assessments indicated little to no impact on the two most eastern beaches (controls). Sequence comparisons of bacterial ribosomal RNA gene hypervariable regions isolated from beach sands located to the east and west of Mobile Bay in Alabama demonstrated that regional drivers account for markedly different bacterial communities. Individual beaches had unique community signatures that persisted over time and exhibited spatial relationships, where community similarity decreased as horizontal distance between samples increased from one to hundreds of meters. In contrast, sequence analyses detected larger temporal and less spatial variation among the water samples. Superimposed upon these beach community distance and time relationships, was increased variability in bacterial community composition from oil hydrocarbon contaminated sands. The increased variability was observed among the core, resident, and transient community members, indicating the occurrence of community-wide impacts rather than solely an overprinting of oil hydrocarbon-degrading bacteria onto otherwise relatively stable sand population structures. Among sequences classified to genus, *Alcanivorax* , *Alteromonas* , *Marinobacter* , *Winogradskyella* , and *Zeaxanthinibacter* exhibited the largest relative abundance increases in oiled sands.

## Introduction

Beach ecosystems represent a natural interface between the land and water where anthropogenic influences may disrupt ecosystem stability and impact microbial communities. In the Gulf of Mexico, beach ecosystems composed of permeable sandy sediments dominate this interface [1]. For many coastal areas including the Gulf of Mexico, sandy sediments extend far out into the ocean, where they might account for more than 70% of the Earth’s shallow seafloor [1]. Microbial biofilms cover the sand particles in these coastal systems [2] and function as three-dimensional biocatalytic filters for the recycling of nutrients throughout the sandy interface. Equally important, beach ecosystems are intimately tied to human interests. Beaches can profoundly impact local and regional economies [3] where they serve as the primary location of physical interaction between humans and the marine environment with attendant impacts upon human health and community vitality.

Water quality monitoring at thousands of marine beaches across the U.S. focuses on the detection of bacterial indicators for human health risks (e.g. [4]), but does not include collection of information about indigenous beach bacterial communities, which makes it difficult to assess disturbance impacts in the beach environment [2,5]. Recently, a few studies employing high-throughput sequencing of 16S rRNA genes from sandy coastal sediments demonstrated that the phyla *Bacteroidetes* and *Planctomycetes* and the class *Gammaproteobacteria* typically represent high abundance taxa in these systems [5,6], confirming earlier reports that employed lower resolution methods [2,7,8]. Despite frequent inundation by seawater through coastal sands, analysis of the bacterial communities of the overlying water column, pore water, and sand biofilm revealed that each environment harbors a unique community [5]. Sand biofilm communities tend to be more diverse than the overlying water column and exhibit temporal variation that does not necessarily coincide with the seasonal cycles observed in nearshore waters [5,9]. Instead, shifts in sandy sediment bacterial communities correlate with several environmental parameters including wave energy [10], organic carbon [11], and primary productivity [5]. However these studies did not explicitly examine the range of temporal and spatial variation necessary to investigate bacterial community stability or disturbance impacts in sandy sediment ecosystems.

The beach ecosystems along the northern Gulf Coast underwent a major disturbance in the months following the blowout of the Deepwater Horizon drilling rig and its release of the equivalent of 4.9 million barrels of oil, 4.1 million of which was released into the coastal ocean [12]. Beginning in mid-June, large quantities of oil and its constituents sporadically washed ashore over a two- to three-month period and were subsequently buried in the nearshore sandy sediments [6,13], bringing enormous amounts of allochthonous carbon to beach ecosystems. Hundreds of bacterial taxa, many of which have been isolated from contaminated sandy sediments, can decompose a variety oil hydrocarbons [14–16] and rapidly proliferate in the presence of oil [6,14,17,18], but the *in situ* impacts on microbial communities from increased oil hydrocarbons in the coastal beach environment remain relatively unknown [6]. Hypothetically, a large, pulsed disturbance similar to that caused by the Macondo Prospect reservoir blowout or any complex hydrocarbon deposition could have major ramifications in coastal environments for the entire bacterial community rather than just for those organisms capable of using the introduced carbon mixture. As resource pools shift following the inundation of large quantities of allochthonous carbon, species reactions to altered nutrient availability could significantly shift community composition.

Instead of focusing on individual taxa, this study explores changes in the entire community structure as a response variable to environmental stressors such as oil. As the direct impacts from oiling diminish following significant breakdown of hydrocarbons, community structure change or variability may reflect more long-term or system-wide impacts from ecosystem disruption. To investigate the response of bacterial communities to sporadic oil exposure in nearshore waters, we used 454 massively-parallel tag sequencing targeting the V6-V4 region of the 16S rRNA gene to profile bacterial community compositions at seven beaches along the northern Gulf Coast stretching from Bay St. Louis, MS to St. George Island, FL USA. Analysis of bacterial community variation in space and time between two non-oiled beaches provided a framework for predicting normal levels of variation in uncontaminated sand ecosystems. By relating the extent of beach oiling at each of the five oiled and the two non-oiled beaches to changes in microbial community structure, we demonstrated the feasibility of using microbial community structure as an indicator of disturbance events in coastal environments.

## Materials and Methods

### Ethics Statement

All samples collected within territories governed by the United States Department of the Interior’s National Park Service were collected in accordance with the details outlined in assigned study GUIS-00142, permit # GUIS-2010-SCI-0044.

### Study sites and sampling procedures

**Figure 1:**
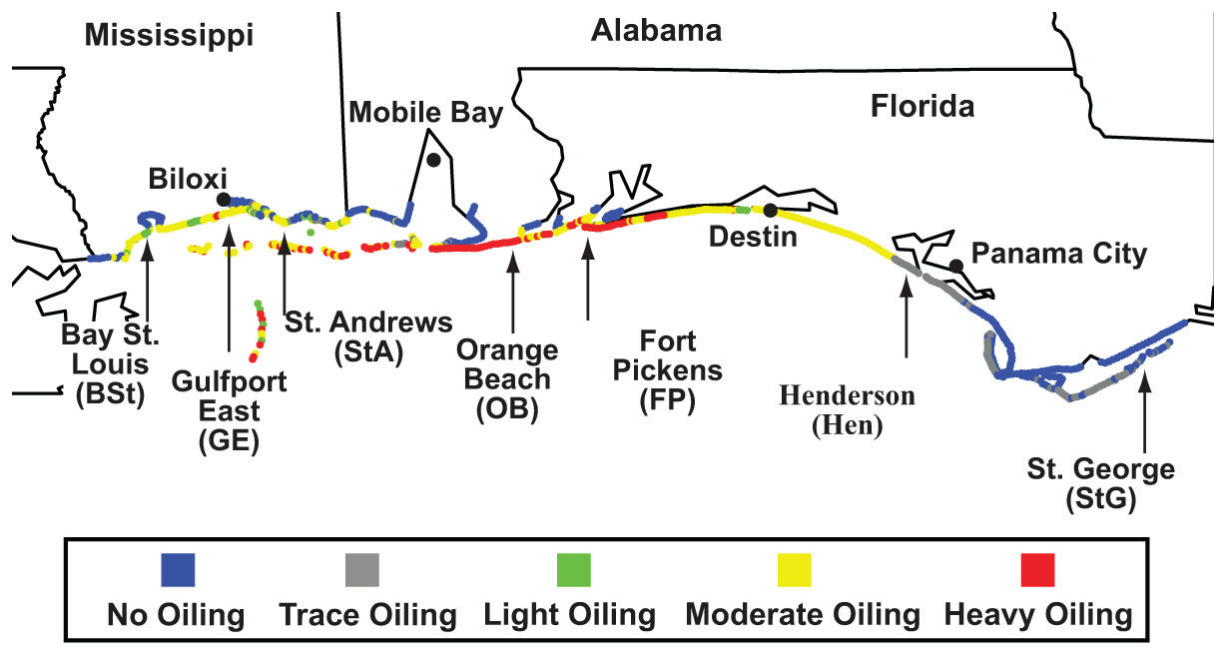
Northern Gulf Coast map indicating the location (arrows) of sampled beaches. Shoreline is colored-coded based on maps from the qualitative extent of beach oiling dataset (April, 2011) from the NOAA Environmental Response Management Application (ERMA^®^) based on NOAA’s shoreline cleanup and assessment technique. See www.geoplatform.gov/gulfresponse for assessment details.

Surface sand and surface surf zone water samples were collected from seven beaches in the southeast United States along the Gulf Coast in Mississippi, Alabama, and Florida. The beach locations were Bay St. Louis (BSt, 30°17'54″ N, 89°20'10″ W), Gulfport East (GE, 30°22'53″ N, 89°01'38″ W) and St. Andrews (StA, 30°20'34″ N, 88°42'29″ W) in Mississippi, Orange (OB, Cotton Bayou Beach area, 30°16'54″ N, 87°41'17″ W) in Alabama, and Fort Pickens (FP, Langdon Beach area, 30°19'01″ N, 87°15'34″ W), Henderson (Hen, 30°22'59″ N, 86°26'34″ W), and St. George Island (StG, 29°41'22″ N, 84°46'59″ W) in Florida (See Figure 1 for sample map). Three environments were sampled at each beach: surf zone surface water, submerged surface sand (intertidal sand located below water in the surf zone), and exposed surface sand (wet intertidal sand located at the high point of wave action on the beach face). For both the submerged and exposed sand environments, we chose three different sampling sites ~100 m apart (labeled 1-3) and collected three 15 ml (by volume) samples ~1 m apart (labeled A-C) from each site. For the water environment, we again selected three sampling sites ~100 m apart, but collected only one 500 ml grab sample per site. All described samples were collected during four sampling periods: June 13-16, 2010, August 8-10, 2010, September 19-22, 2010, and November 15-18, 2010, except for St. George, which we were unable to return to during the August period. Following collection in the field, the sand samples were stored on ice between 2–28 hours during the sampling expedition and then on dry ice before being shipped to the lab for further processing. See Table S1 for details on conditions during the sample collection. In the lab, sand was stored at -80 °C prior to DNA extraction procedures. Following collection of the water, two 200 ml aliquots from each sample were filtered in the field with a Mityvac® vacuum hand pump (Lincoln Industrial Corp., MO, USA) through 0.22-µM-pore-size mixed cellulose ester filters (47-mm diameter; Millipore, Billerica, MA, USA) and stored in 2 ml collection tubes on ice prior to shipping on dry ice. Once in the lab, the filters were stored at -80 °C prior to DNA extraction. In addition, a single surface exposed sand sample for hydrocarbon analysis was collected in 120 ml glass jars at each site at the location of the samples collected for microbial community analysis.

### DNA extraction

To extract DNA from the sand, samples were removed from the freezer, thawed, and one gram of sand was transferred to an extraction tube. Extraction procedures continued according the manufacturers instructions for the MP Biomedicals FastDNA Spin Kit for Soil (Solon, OH, USA), except that during the cell lysis step, a Mini Beadbeater 8 was used with the “homogenize” speed for 1 minute. DNA was extracted from the filtered water samples using the MP Biomedicals FastDNA Spin Kit for Soil, with modifications as described previously [19].

### Amplicon library construction

We constructed amplicon libraries for the bacterial 16S rRNA gene V6 through V4 domains (amplification in the reverse DNA strand orientation). Amplification fusion primers contain either the A or B 454 Titanium amplicon adapter for GS-FLX sequencing (Roche Diagnostics, Indianapolis, IN, USA) followed by a 5 nt multiplex identifier (MID) and ending with the 16S specific sequence. The 16S sequences used were 518F, 5’ CCAGCAGCYGCGGTAAN and 1064R, 5’ CGACRRCCATGCANCACCT. The MID is present in both fusion primers. MIDs differ by at least two bases and contain no homopolymers. The 518F/1064R fusion primer set was designed to capture over 95% of known eubacterial diversity. The primers match 16S rRNA genes from 50 to 100% of the members of all known phyla in our reference database based on the SILVA 106 release [20] with the exception of a few phyla containing little known diversity, such as OP 11 and SR1.

The polymerase chain reaction mixture contained 1X Platinum HiFi Taq polymerase buffer, 1.6 units Platinum HiFi polymerase (Life Technologies, Carlsbad, CA USA), 3.7 mM MgSO_4_, 200 µM dNTPs (PurePeak polymerization mix, Thermo, Fisher, E. Providence, RI, USA), and 50 nM combined primers. 5-25 ng of sample DNA was added to a master mix to a final volume of 100 µl and this was divided into three replicate 33 µl reactions. We included a no-template negative control for each MID. Conditions were an initial denaturation at 94^o^C for 3 minutes; 30 cycles of 94^o^C for 30 seconds, 60^o^C for 45 seconds, and 72^o^C for 1 minute; and a final extension at 72^o^C for 2 minutes using an Applied Biosystems 2720 or 9700 cycler (Life Technologies). The three replicates were pooled and we checked 1 µl of the pool and the negative control on a LabChip GX (Caliper, Hopkinton, MA USA). We cleaned the reaction and removed products under 300 bp using Ampure beads at 0.75X volume (Beckman Coulter, Brea CA). The final products were resuspended in 100 µl of 10mM Tris-EDTA, quantified using PicoGreen Quant-IT assay (Life Technologies), and assayed on a Bioanalyzer DNA1000 chip (Agilent Technologies, Santa Clara, CA, USA).

### 454 sequencing and signal processing

We pooled up to 40 amplicon libraries prior to emulsion PCR. The emPCR, enrichment, and sequencing were done according to current Roche Titanium amplicon sequencing protocols (Lib-A emPCR reagents, XLR sequencing reagents, two region PicoTitre plate). A typical sequencing run generates 10-20,000 tags per library. Image processing and signal calling were done using the Roche amplicon processing pipeline (version 2.53) with recursive phase correction algorithm to maximize the number of long reads that passed quality control procedures. In total, pyrosequencing was carried out on 127 samples: 46 exposed sand, 46 submerged sand, and 35 water. See Table 1 for the list of samples used in pyrotag sequencing.

**Table 1:** Sample Sequencing Plan.

**Beach**	**Sand** ^a^				**Water**			
	Jun^b^	Aug	Sep	Nov	Jun	Aug	Sep	Nov
**Bay St. Louis**	1A, 2A^c^	1A+2A^d^	1A+2A	1A+2A	2^e^	2	2	2
**Gulfport East**	1A, 1B, 1C, 2A, 2B, 2C, 3A	1A+2A	1A+2A	1A+2A	1, 2, 3	2	2	2
**St. Andrews**	1A, 2A	1A, 2A, 3A	1A+2A	1A+2A	1, 2	1, 2	2	2
**Orange, Beach**	1A, 2A	1A+2A	1A+2A	1A+2A	1, 2	2	2	2
**Fort Pickens**	1A, 1B, 1C, 2A, 2B, 2C, 3A	1A+2A	1A+2A	1A+2A	1, 2, 3	2	2	2
**Henderson**	1A, 2A	1A+2A	1A+2A	1A+2A	2	2	2	2
**St. George**	1A, 2A	N/A	1A+2A	1A+2A	1, 2	N/A	2	2

^a^ Sequencing plan for both exposed and submerged sands

^b^ Month abbreviations: Jun = June, Aug = August, Sep = September, Nov = November.

^c^ At each beach, three sand samples (A, B, C) were collected at each of three sites (1, 2, 3) located approximately 100 m apart.

^d^ 1A+2A, indicates the 1A sample and 2A sample were combined in equal weight proportions prior to DNA extraction.

^e^ At each beach, three water samples (1, 2, 3) were collected approximately 100 m apart

### Bioinformatics Processing

Pyrosequencing reads were quality-filtered by removing reads that did not have exact matches to 1064R and the MID, that contained an ambiguous base (N), that lacked the reverse-complement of conserved bases 5’-TGGGCGTAAAG-3’ (position 565F in *E. coli*) allowing 2 mismatches, or that had an average quality score less than 30 [21]. Reads were trimmed after the reverse-complemented 565F conserved sequence. Chimeras were removed using UChime [22], combining both the *de novo* and reference database (ChimeraSlayer GOLD) modes. Taxonomy was assigned using GAST [23] and the data uploaded to the Visualization and Analysis of Microbial Population Structures website (VAMPS: http://vamps.mbl.edu) for analysis. OTU clustering was performed using UClust [24] at 97% sequence identity to minimize contribution of sequencing errors toward cluster inflation. All trimmed and filtered sequence tag data and taxonomy are available on VAMPS under the SLM_GCB_Bv6v4 project name, and the sequence files are also available at the National Center for Biotechnology Information (NCBI) Sequence Read Archive, Bioproject PRJNA208242.

### Hydrocarbon Analysis

Pace Analytical Services, Inc. (St. Rose, LA, USA) carried out the oil hydrocarbon analyses. All results conform to the National Environmental Laboratory Accreditation Conference (NELAC) standards. Oil hydrocarbon concentrations (83 compounds for each sample) were quantified with the EPA 8270 method using a gas chromatography-mass spectrometry (GC-MS) technique for semivolatile organic compounds and polyaromatic hydrocarbons. Unknown oil hydrocarbons totals were summed across all detected compounds with C8-C40 compositions.

### Community, OTU, & Taxonomic Statistical Analyses

Unless stated otherwise, all bacterial community analyses were carried out with the sequences grouped as OTUs, defined as sequences sharing ≥97% identity (OTU clustering described above). Analyses were carried out with the OTU counts for each sample normalized to the total bacterial sequences in that sample. Excluding the within-site, within-beach analyses, all other analyses were carried out with a single set of community composition data for each environment-beach-date combination. For sample dates where multiple samples were sequenced at a single beach, the mean relative abundance of each OTU was calculated. Community similarity among samples was calculated with the Morisita-Horn index [25] using the vegan package [26] vegdist() function in the R statistics package (R version 2.13.1 [27]). Compared to the Bray-Curtis index, another commonly used similarity measure in ecological studies, Morisita-Horn is less sensitive to differences in richness and tends to report increased similarity among samples [28]. Our dataset contained many large differences in community composition and variable richness among samples (data not shown), therefore we chose to use the Morisita-Horn index to compare sample similarities. Dendrograms were calculated using the hclust() function with average-linkage clustering in the R statistics package (R version 2.13.1 [27]).

Analysis of Similarity (ANOSIM [29]) calculations were implemented to test hypotheses related to within-group sample similarity being greater than among-group sample similarity. ANOSIM is a nonparametric technique that allows statistical comparisons for multivariate data sets. All ANOSIMs were run with 10,000 permutations. For comparisons of sample group community similarity variability, the Student’s t-test was implemented.

Core, resident, and transient OTUs were arbitrarily defined as those occurring in ≥75%, 25-75%, and <25% of all exposed sand or water samples. OTUs recovered only once (i.e. occurring as a singleton) were not included in these categorical analyses. For all categorical analyses, the sequencing effort between samples was normalized within each environment. OTUs were drawn randomly from each sample with 6250 draws from the sand samples and 5775 draws from the water samples, which corresponded to the lowest quality-controlled sequence count in each environmental set. Randomization was carried out using the sample() function the R statistics package (R version 2.13.1 [27]). The sub-sampled data was used for all subsequent analyses involving the core, resident, and transient community data.

The SIMPER algorithm [29] in the statistical package PRIMER v6, (PRIMER-E Ltd. [30]) was used to identify the taxa most contributing to the community composition differences between oiled and non-oiled samples/beaches. The SIMPER method ranks and lists the contribution of each defined taxa to the within-group similarity and among-group dissimilarity so that those taxa most distinguishing the a priori defined groups may be identified. A two-way crossed SIMPER design was used to control for the differences between samples collected to the east and west of Mobile Bay because of the strong community patterns related to this geographic division. To remain consistent with previous analyses, Morisita-Horn sample distance matrices calculated in R were imported and used in the SIMPER analyses.

## Results

### Hydrocarbon measurements and estimated accumulation at beaches

Qualitative estimates for the extent of beach oiling as reported by NOAA’s cleanup and assessment technique suggested that Bay St. Louis had light oiling, Gulfport East and St. Andrews had moderate oiling, Orange, Beach and Fort Pickens had heavy oiling and Henderson and St. George had either trace or no oiling (Figure 1). Each of the five most westerly beaches also had detectable levels of oil hydrocarbons in at least one sample (Table 2). Since the Henderson and St. George sites had no sample containing measurable oil hydrocarbons, they served as control sites for the study. The hydrocarbon measurements also indicated that only Gulfport East had measurable oil hydrocarbon concentrations in more than one sampling period (Table 2; June, August, and September) and that the Orange, Beach June sample, the only sample collected when visible oil was washing ashore, had a concentration 1000 times greater than any other sample (Table 2).

**Table 2:** Petroleum hydrocarbon concentration (µg compound / kg sand).

Beach - Month^a^	Fluoranthene	Fluorene	2-Methylnaphthalene	Naphthalne	Phenanthrene	Pyrene	Benzo[a]pyrene	Unknown ≥10C
BSt - Jun	**3.0**	-	-	-	-	**2.3**	**2.3**	-
GE - Jun	-	-	-	-	-	-	**6.3**	-
GE - Aug	-	**1.7**	**1.4**	**1.1**	-	-	-	-
GE - Sep	-	-	-	-	-	-	**3.0**	-
StA - Aug	-	-	-	-	-	-	**30.8**	-
OB - Jun	**8.6**	-	-	-	**89.4**	**5.3**	-	2.14*10^4^
FP - Jun	-	-	-	-	-	-	**2.1**	-

^a^ Beach abbreviations: BSt = Bay St. Louis, GE = Gulfport East, StA = St. Andrews, OB = Orange, Beach, FP = Fort Pickens, Hen = Henderson, StG = St. George. Month abbreviations: Jun = June, Aug = August, Sep = September, Nov = November. Only beach – month combinations where petroleum hydrocarbons were detected are shown. The no-detect beach – month combinations include: BSt – Aug, BSt – Sep, BSt – Nov, GE – Nov, StA – Jun, StA – Sep, StA – Nov, OB – Aug, OB – Sep, OB – Nov, FP – Aug, FP – Sep, FP – Nov, Hen – Jun, Hen – Aug, Hen – Sep, Hen – Nov, StG – Jun, StG – Sep, StG – Nov.

### Bacterial taxa of coastal sands and nearshore water

**Figure 2:**
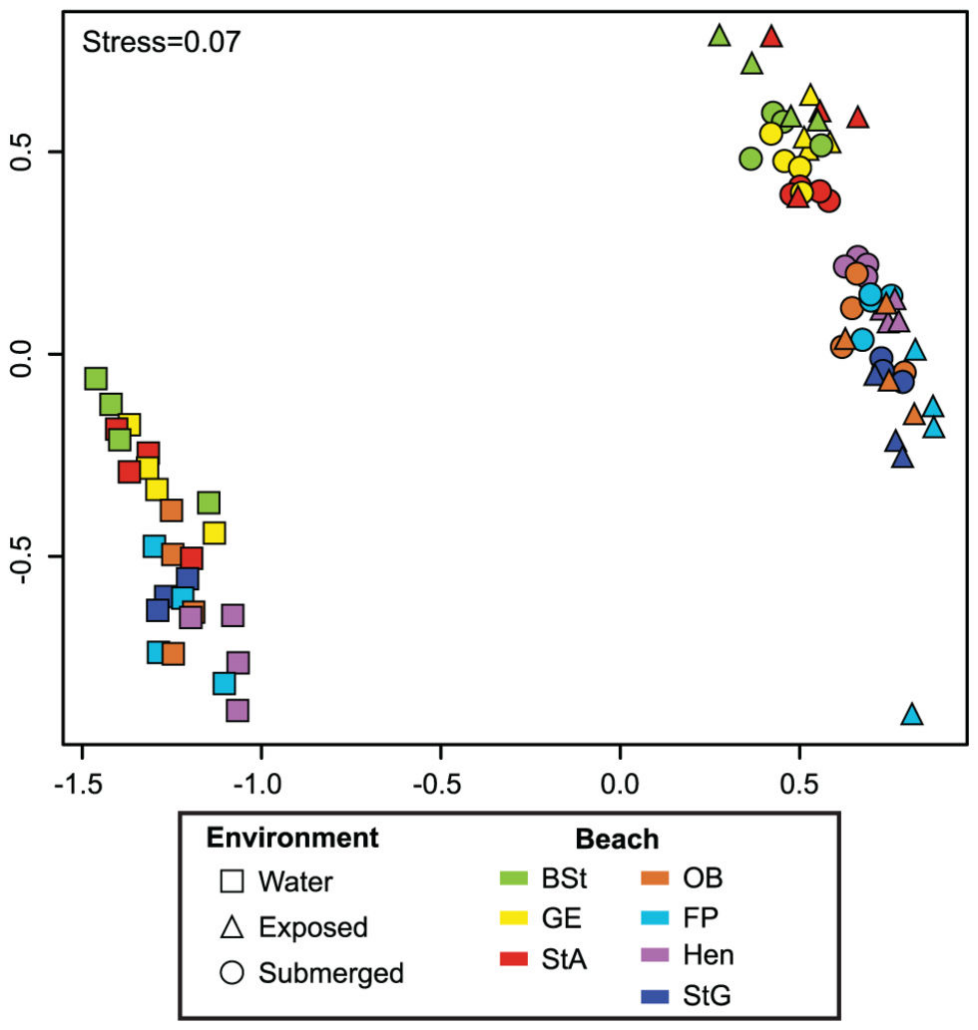
Non-metric multidimensional scaling (NMDS) plot of bacterial community composition among all sand and water samples. The NMDS is based on OTU community similarity (Morisita-Horn) among samples from all exposed and submerged sand and water samples. The average OTU composition for all beaches with multiple sequenced samples from the same date is represented by a single sample point.

Pyrosequencing of environmental DNA extracted from the sand and water samples generated 1,583,119 high quality bacterial rRNA gene pyrotags spanning the V4 to V6 region. The bacterial composition of surface water communities across all seven beaches and four sampling dates clustered together in a non-metric multidimensional scaling plot (NMDS) plot, to the exclusion of both the submerged and exposed beach sand communities (Figure 2). The two sand environments were found to have relatively similar (low R value), but significantly different community structures (Figure 2, ANOSIM R=0.202, p=0.001; comparison of exposed to submerged sand communities while controlling for region, east/west of Mobile Bay).

**Figure 3:**
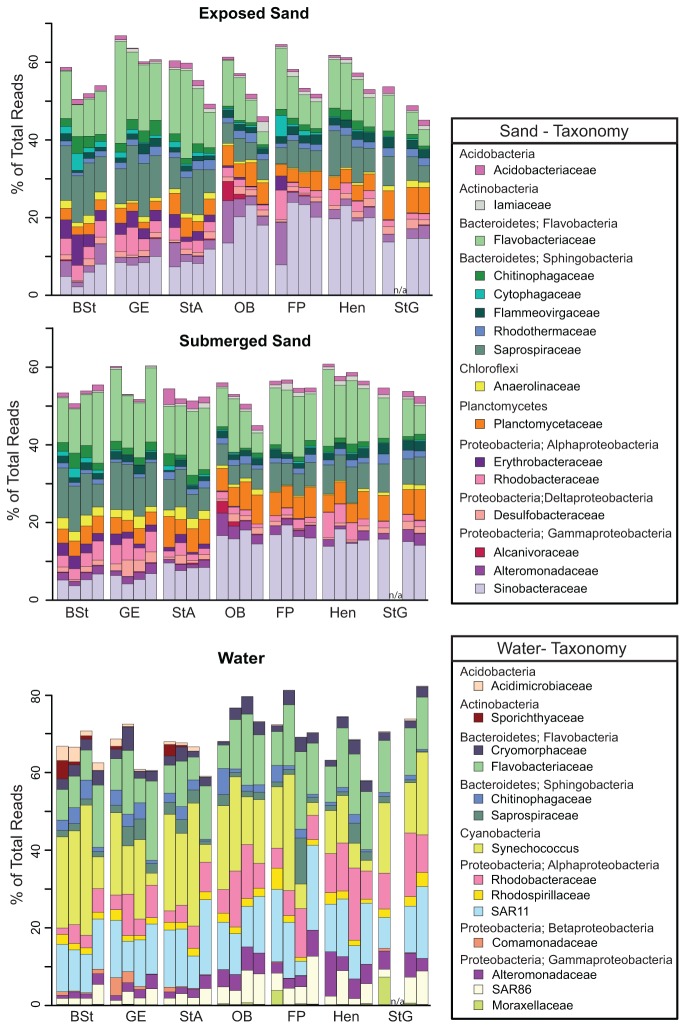
Family-based taxonomic representation of each bacterial community. Families were only included in the plot when they were among the top-5 most abundant families in at least one of the samples. Sand and water family rankings were treated independently. The average family composition is represented for all beach – date combinations containing multiple sequenced samples.

Of the 77.3% of total sand pyrotags classified to family or more refined taxonomic levels, the *Flavobacteriaceae* and *Saprospiraceae* (phylum *Bacteroidetes*), the *Planctomycetaceae* (phylum *Planctomycetes*), and the *Sinobacteraceae* (phylum *Proteobacteria*, class *Gammaproteobacteria*) were the most abundant (Figure 3). Among the genus level assignments (48.4% of total sand pyrotags classified to genus), *Zeaxanthinibacter* (*Bacteroidetes*), *Haliscomenobacter* (*Bacteroidetes*), and *Erythrobacter* (*Alphaproteobacteria*) were most abundant in the exposed samples (14.5% of total bacterial pyrotags), while *Zeaxanthinibacter* , *Haliscomenobacter* , and *Blastopirellula* (*Planctomycetes*) were most abundant in the submerged samples (14.3% of total bacterial pyrotags; See Table S2 for details). We also observed a shift from a *Proteobacteria* (mainly *Gammaproteobacteria*) dominated community in the sands east of Mobile Bay (OB, FP, Hen, StG) to a community generally dominated by *Bacteroidetes* in the sands west of the bay (BSt, GE, StA; Figure 3).

In the water samples, 84.7% of all pyrotags were classified to family or more refined taxonomic levels. Three families, *Flavobacteriaceae*, *Rhodobacteraceae*, and SAR11, as well as the genus *Synechococcus* , dominated these bacterial communities with only *Flavobacteriaceae* representing a dominant taxon in both the sand and water environments (Figure 3). Among the pyrotags classified to genus (56.5% of all water pyrotags), *Synechococcus* (*Cyanobacteria*)*, Pelagibacter* (SAR11) and *Owenweeksia* (*Bacteroidetes*) were the most abundant, together making up 30.7% of the total bacterial pyrotags in the water samples (see Table S2 for details). We also recovered a relatively high number of sequences from the *Comamonadaceae* family of *Betaproteobacteria* in the Mississippi Sound water samples, particularly at the Gulfport East Beach site (Figure 3).

### Spatial Variability among Bacterial Communities

**Figure 4:**
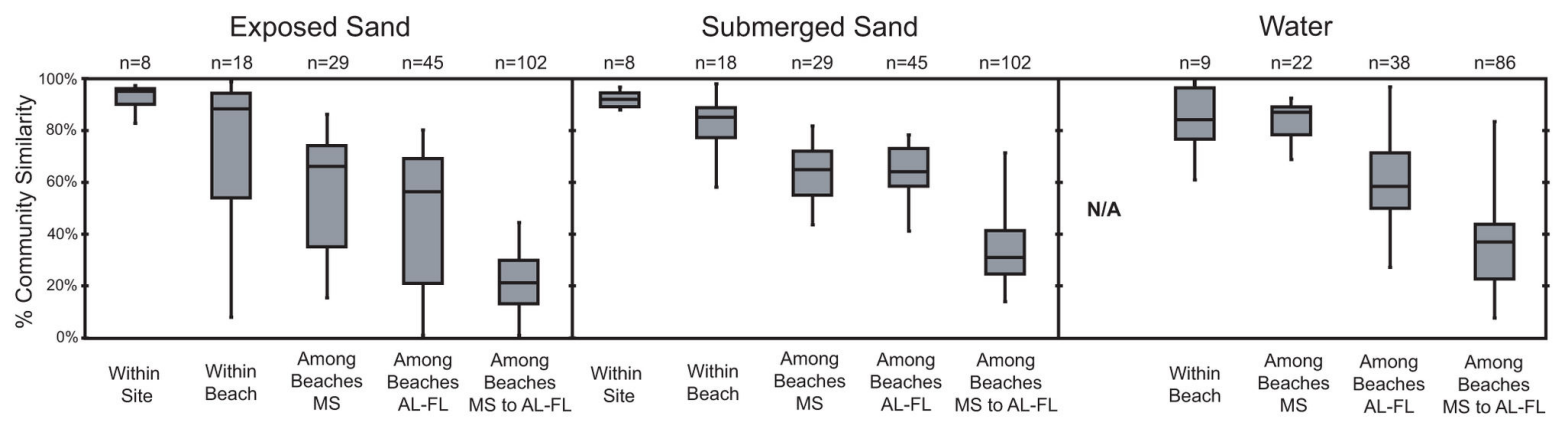
Bacterial community similarity comparison among groups based on spatial relationships. Box plots are based on pairwise comparisons of Morisita-Horn bacterial community similarity within each sample group. The box indicates the 25% quartile, median, and 75% quartile, and the maximum and minimum values are indicated at the tips. Within-site samples were collected approximately 1 meter apart; within-beach samples were collected among three sample sites (~100 meters apart) at each beach. The number of sample comparisons (n) used in constructing the box plot is listed.

**Figure 5:**
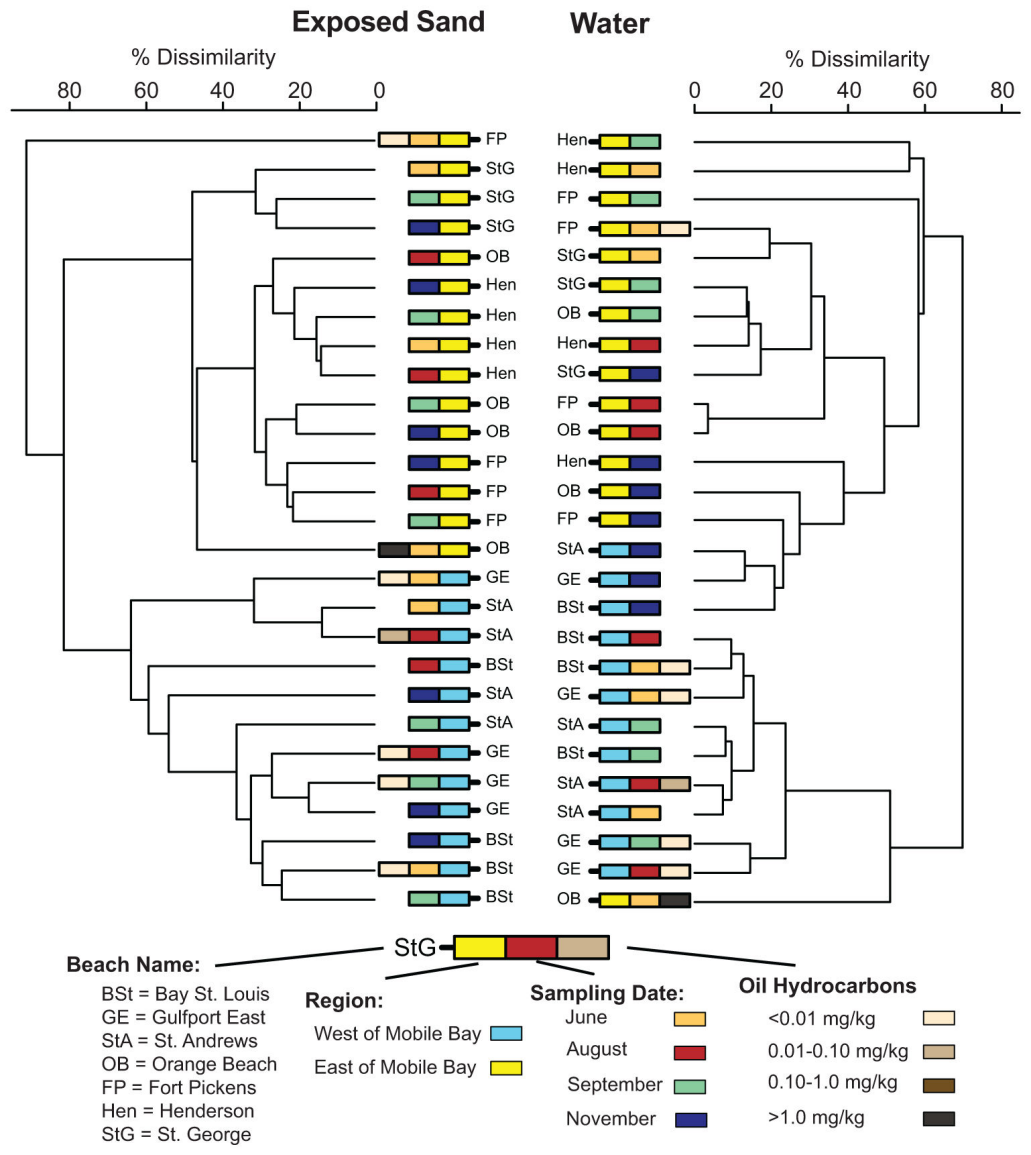
Dendrogram illustrating the bacterial community composition relationships among samples and select sample characteristics. An average-group linkage dendrogram is presented to illustrate the bacterial community composition relationships among the exposed sand (left) and water (right) samples. The mean OTU composition was used to represent beaches with multiple sequenced samples from the same date. Sample features are indicated with colored boxes according to the key. For example, the first rectangle next to the beach name represents samples collected from either west of Mobile Bay (blue) or east of Mobile Bay (yellow).

At nearly all spatial scales, the submerged sand communities exhibited greater community composition similarity and less community variation than the exposed communities (Figure 4). In both the exposed and submerged sand environments, community similarity scaled with distance between collection points, where samples taken within a few meters (within site) were more similar than those samples taken within 100s of meters at the same beach (within beach), which in turn were more similar than samples from >1 km apart (among beach; Figure 4). Cluster analysis revealed a large regional effect that distinguished community composition patterns according to sample location, either east or west of Mobile Bay (Figure 5; ANOSIM R = 1.0, p-value = 0.0001). However, community composition relationships did not scale with increased distance between beaches (geographic distance vs. community similarity, linear fit R^2^ = 0.20). In the water samples, we also observed a regional effect between samples to the east and west of Mobile Bay (Figure 5; ANOSIM R = 0.65, p-value = 0.0001), although it was much less pronounced than in the sand samples, and exhibited cross-region clustering among the November samples.

### Temporal Variability among Bacterial Communities

**Figure 6:**
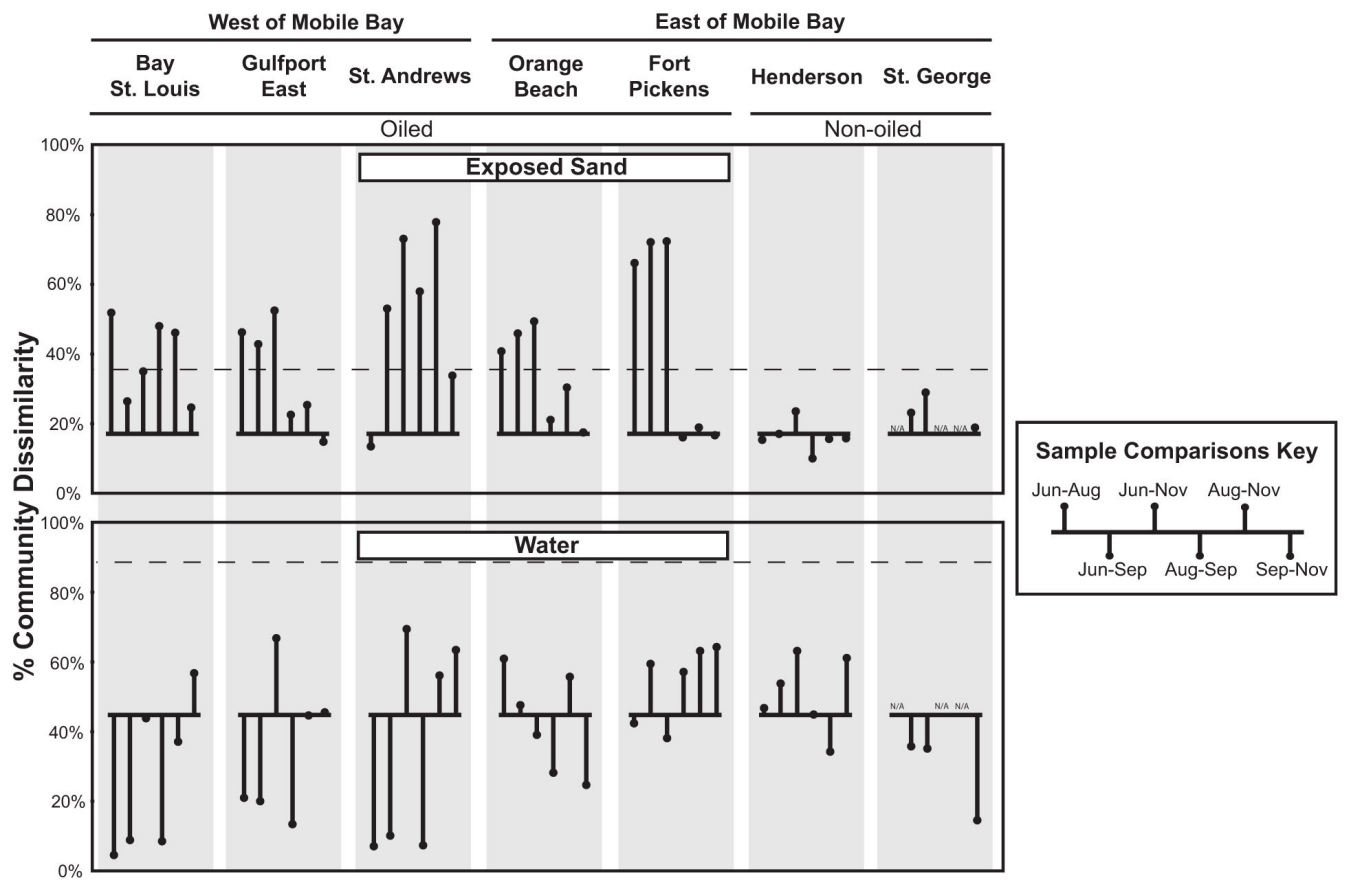
Bacterial community composition dissimilarity variation over time within each beach. Lollipop plots indicate the average Morisita-Horn bacterial community dissimilarity among samples collected over time at each beach. All sample comparisons are indicated (e.g. the June sample is compared to August, September, and November samples). The lollipops originate from a line indicating the mean dissimilarity among samples from the non-oiled beaches (Henderson & St. George). The dashed horizontal line indicates three standard deviations above the mean dissimilarity for the non-oiled beaches.

Since, a general similarity existed between the exposed and submerged sand bacterial communities and our hydrocarbon measurements were conducted only in the exposed sands, we analyzed only the exposed sand communities for all beach oiling and sand microbial community structure comparisons. The stability of the non-oiled beaches (mean community similarity between consecutive timepoints, 83.3%) presented a stark contrast to the large shifts in community composition among consecutive time periods in the oiled beaches (Figure 6). In each oiled beach, the samples from at least one sample period exhibited a mean community change of more than three standard deviations greater than the changes in the non-oiled beaches (Figure 6). Nearly all of the extreme community change values occurred during June and August, which corresponded to the detection of oil hydrocarbons at these beaches (Table 2). However, the sand bacterial communities exhibited weak community composition-based clustering when grouped according to oil hydrocarbon detection (Figure 5; ANOSIM R = 0.38, p-value = 0.04). The final time point comparison (Sep. to Nov.) also did not show a difference in community change between the oiled (78.5% mean community similarity) and non-oiled beaches (82.6% mean community similarity; t-test p-value = 0.53). In contrast to the sand communities, the water communities exhibited larger temporal community variation, with generally higher variability among the later sampling dates (Figure 6). This large variability occurred at both the oiled and non-oiled beaches, and none of oiled beach water samples exceeded three standard deviations greater than the changes in the non-oiled beaches.

### Community Composition: Core, Resident, & Transient

To test whether the presence of new or low abundance taxa/OTUs increasing in relative abundance and/or the changes in the relative abundance of common taxa/OTUs accounted for changes in the oiled beach communities, we divided OTUs from each environment into three occurrence based categories: core, resident, and transient. For the exposed sand and water samples respectively, 26 and 62 OTUs corresponded to core, 1587 and 1306 corresponded to resident, and 22,057 and 18,043 corresponded to transient.

In the core and resident sand communities, the non-oiled beach samples clustered by beach, while the oiled beaches were less stable (Figure S1). In the transient sand communities, all samples significantly clustered by beach (ANOSIM R = 0.90 p = 0.0001). Only the Orange, Beach and Fort Pickens samples and all three MS beach June samples (oiling period) did not strictly follow this pattern. The water core, resident, and transient communities weakly clustered by beach over time (ANOSIM R = 0.43-0.45, p = 0.0001 for all occurrence categories), and only the resident community exhibited any, albeit a very weak, relationship to the presence of oil hydrocarbons (S Figure 1; ANOSIM R = 0.21, p = 0.01).

**Figure 7:**
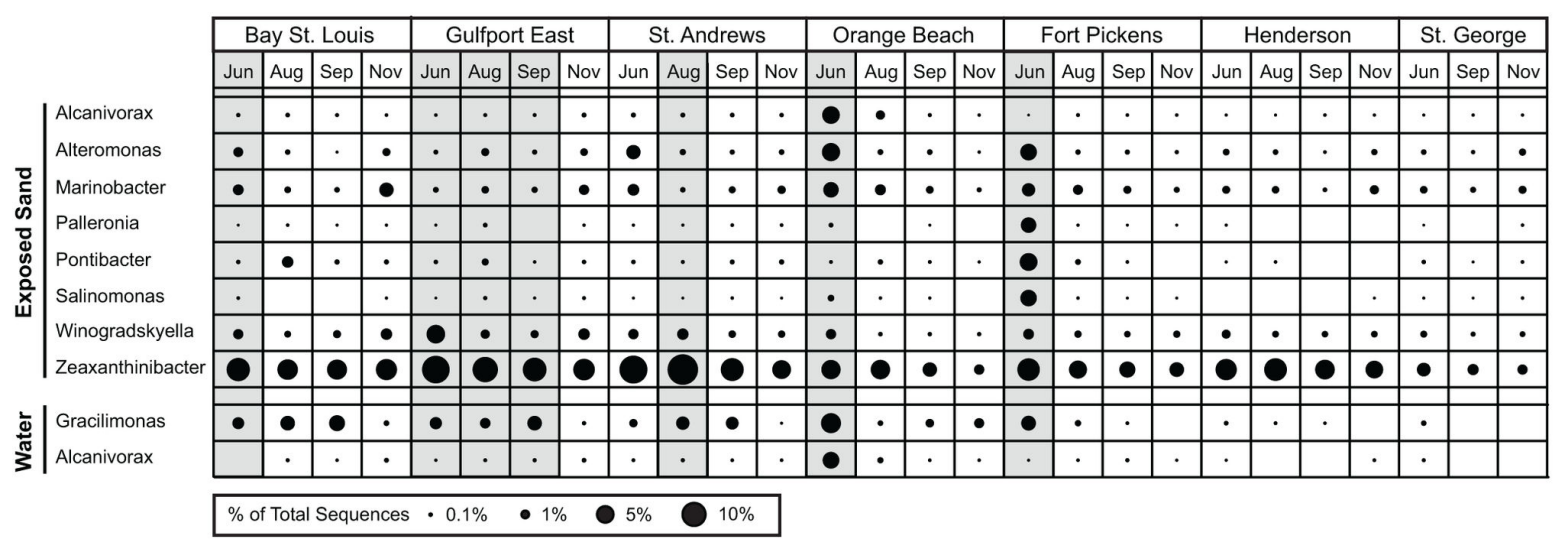
A relative abundance comparison of taxa most contributing to the differences between oiled and non-oiled samples. A balloon plot indicates the relative abundance of each taxon among samples. Taxa were included only if they were among the top 10 taxa most distinguishing the community compositions of oiled and non-oiled beaches and were positively related to the presence of oil hydrocarbons (SIMPER algorithm). Samples shaded gray indicate oil hydrocarbons were detected in that sample.

Since community composition patterns related to beach oiling were present in each of the occurrence-based community groups, we used the SIMPER algorithm [29] to identify taxa at the genus level that most contributed to defining patterns among oiled and non-oiled samples. Among the core community OTUs, the *Zeaxanthinibacter* and *Marinobacter* genera exhibited the greatest relative increase in the oiled samples (Figure 7), while in the resident communities, *Alteromonas* and *Pontibacter* and in the transient communities *Alcanivorax* and *Salinimonas* exhibited a similar relationship. In particular, *Alcanivorax* , *Alteromonas* , and *Marinobacter* showed large increases in the Orange, Beach June sample that we collected when oil was washing ashore, while the other genera generally increased in the beach samples that lacked visible evidence of oil. In the water communities, no consistent shifts for core community taxa corresponded to the presence of oil hydrocarbons in beach sands. In contrast, large increases of *Gracilimonas* and *Alcanivorax* in the resident community correlated with detection of oil hydrocarbons with *Alcanivorax* accounting for the major differences in the Orange, Beach June sample (Figure 7).

## Discussion

In this study we examined microbial community structure as a measure of the impact from oil hydrocarbons on beach ecosystems. To accomplish this goal, we characterized surface sand and nearshore water communities at coastal beaches, delineated spatial and temporal patterns at beaches that were oiled between June and November 2010 and at those that did not receive appreciable oiling during that period, and identified taxa changes driving the observed community patterns. Although the Macondo Reservoir blowout provided a defined event that resulted in wide-scale beach oiling [6,13], we were interested only in understanding the response of microbial communities to an oil hydrocarbon pulsed disturbance, not the response related specifically to an oil hydrocarbon source. Thus, any oil hydrocarbons washing ashore during our sampling period were included as part of the analyses.

### General characteristics of sandy beach microbial communities

Previous studies report that despite continuous flushing of ocean waters through coastal beach sands, the bacterial community composition of overlying waters differs from the community present in sands [5] including significant differences related to sand depth [5,9,31]. The results from our bacterial community sequencing effort support these previous findings (Figure 2), but include a greater resolution across spatial scales. Decreased contact with the water and therefore increased desiccation and decreased nutrient exchange differentiates exposed sand and submerged sand. However, our results identify similar community compositions for surface exposed and submerged sand communities, likely reflecting the minimal slope to most Gulf Coast beaches, which allows for large tidal zones that frequently move sandy sediments across the beach face [32]. It also suggests sands receiving periodic wetting events do not undergo drastic changes to community structure during short-term dry periods. Further investigation is needed to understand whether the nutrient-starved beach backshore microbial communities, which rarely contact ocean water, harbor a distinct community composition from sands in the intertidal or subtidal zones.

The results from our taxonomic characterization of the community (Figure 3) generally agree with previous results that suggest *Proteobacteria* (*Gammaproteobacteria*), *Planctomycetes*, and *Bacteroidetes* taxa dominate coastal sands [2,5,33,34]. Although not examined here in detail, the difference in environmental conditions in the Mississippi Sound estuarine system (west of Mobile Bay), which is protected by a series of barrier islands and receives large nutrient loads, from the remainder of the Gulf Coast (east of Mobile Bay) may partition this region into two environmental provinces that select for similar but distinct community compositions. A more thorough characterization of the beach sands along the remainder of the Gulf Coast, including the Mississippi barrier islands would be needed to further delineate microbial provinces in this region, which could contribute to defining management areas according to ecosystem drivers.

The high abundance of *Synechococcus* , *Pelagibacter*, roseobacters, SAR86, and marine members of *Flavobacteriaceae* in our water samples supports many previous findings of bacteria in coastal waters (e.g. [35,36]). However, the recovery of a large number of sequences from the *Comamonadaceae* family of *Betaproteobacteria* in the Mississippi water samples, particularly at the Gulfport East Beach site (Figure 3) indicates a strong terrestrial influence. *Betaproteobacteria* are not typically abundant in ocean waters [36,37], but to our knowledge few studies have characterized the bacterial community in near shore surf zone waters. The Gulfport East Beach site is very near (10s of meters) a stormwater outfall, which carries freshwater runoff to the ocean. Freshwaters including estuaries harbor greater numbers of *Betaproteobacteria* mainly composed of *Comamonadaceae* taxa [37,38], and could be the source in these surf zone waters.

### Bacterial community variability as a measure of disturbance impact

A disturbance may be defined as ‘any relatively discrete event in time that disrupts ecosystem, community, or population structure and changes resource pools, substrate availability, or the physical environment’ [39]. The diverse origins of disturbance events cause large variation in the kind and magnitude of ecosystem response. Changes in system variability as a measure of disturbance impact has recently had a resurgence in popularity as researchers seek universal measures of disturbance effects on ecosystems and leading indicators for ecosystem regime shifts [40,41]. Microbes as direct and rapid responders to changes in the chemical cues of their surrounding environment may serve as acute sensors of environmental change and thus seem a natural fit for the measurement of ecosystem impacts from disturbances.

The relationship between a disturbance and changes in microbial community structure requires detailed information about the undisturbed community and its variability in space or over time. In previous studies, surface sand microbial community change correlated with changes in organic carbon (Polymenakou et al., 2005), chlorophyll *a* [9,11], and inorganic nutrient concentrations [42], all of which are influenced by altered wave action and porewater flow [2,9]. Rather than identify the specific environmental conditions that influence community surface sand or nearshore water composition, in this study we attempted to define a range of expected variation from the cumulative effects of environmental factors. This range was set by two beaches, Henderson and St. George, which did not receive significant oiling, but because of their proximity to the oiled beaches experienced similar weather and general environmental conditions.

The high levels of reproducibility among samples 1 m apart (within-site, Figure 4) and among samples 100 m apart at each beach (within-beach, Figure 4) allowed us to link changes in community composition or variability to other factors, such as beach oiling. The distinct sand community composition and temporal stability of the non-oiled control beaches, St. George and Henderson (Figure 5, 6 & Figure S1), relative to the oiled beaches provided a reference framework for evaluating the sand communities. In contrast, the greater temporal variability among water samples at all beaches defined a lower level of sensitivity for detecting community patterns related to beach oiling. This is not surprising given that many studies have observed large, seasonally-recurrent community patterns in marine waters [43–46]. In a long-term monitored system, these seasonal community patterns could be bounded and used to identify deviations that reflect impacts from disturbance/pollution effects.

Using the control beaches to establish expected levels of community variability and sample relatedness, we documented significantly increased temporal variability in the sand microbial communities from oiled beaches in the months immediately following the oil release (Figures 5 & 6). By the transition from the September to November sampling period, the change in community composition except at St. Andrews beach, was much lower, reaching levels near those observed for the non-oiled beaches (Figure 6). This was in stark contrast to the large seasonal changes that had occurred over this period as water temperatures had dropped on average by more than 9^°^C (Table S1). Further, when oil reached beaches along the Gulf Coast, it was quickly buried in the supratidal zones of beaches by tidal porewater flow through the beach face [6,13]. With little new oil being delivered to the Gulf of Mexico after the containment in July 2010, the oiling events along coastal beaches were greatly reduced over the next few months [13]. Although changing environmental conditions undoubtedly influenced community composition at each beach, the contrast of the relatively low and consistent magnitude of community change at the non-oiled beaches with the high and inconsistent magnitude of community change at the oiled beaches and the drop in the magnitude of this change as time post beach oiling increased, is consistent with the idea that beach oiling was responsible for the community shifts. We therefore also suggest microbial community variability may be an appropriate indicator of disturbance effects in sandy beaches. It remains to be seen whether the buried oil in these beaches has a prolonged impact on sand community structure or whether this impact manifests as increased community composition variability.

### Composition patterns within communities: core, resident and transient OTUs

Investigations of community structure by categorizing each OTU as either core, resident, or transient, based on its occurrence patterns among samples, corresponds in ecology literature to the core-satellite hypothesis or derivations thereof [47,48] and has been recently discussed for microbial community analysis [45,49]. This classification scheme allowed us to examine whether the observed community shifts related to beach oiling corresponded to an overprinting of oil hydrocarbon degrading taxa onto a relatively stable core community as opposed to composition shifts for the core and/or whole community. Other than those OTUs associated with *Marinobacter* , the core bacterial OTUs in the sand samples did not include known oil-degrading taxa [14–16]; therefore, if beach oiling simply resulted in an overprinting of oil-degrading taxa, then large changes in community composition should have occurred in either the resident or transient communities. Instead, all levels of community categorization reflected beach oiling patterns, where non-oiled beach samples clustered together over time while oiled beaches exhibited less stability (Figure S1). Likewise, OTUs in all three categories contributed to the taxa most related to the community differences observed. Among the core community, *Winogradskyella* , and *Zeaxanthinibacter* , which are genera not known to be oil degraders, exhibited relative increases during periods of beach oiling. In the one sample set collected while oil was visibly washing ashore (OB June), *Alcanivorax* (transient), *Alteromonas* (resident), and *Marinobacter* (core) were largely increased in the community. Kostka and colleagues also observed large increases in the genus *Alcanivorax* , a known degrader of alkanes [15,16], in oiled beach sands at *Pensacola* Beach, FL following the Macondo blowout, and its abundance largely drove the community composition patterns they observed across samples [6]. Members of the genus *Marinobacter* are also commonly identified as petroleum hydrocarbon degraders [15,16], while the genus *Alteromonas* only occasionally has been associated with oil hydrocarbon enrichments [17]. The increase in relative community contribution of these known oil-degrading genera lends further evidence to the idea that community shifts in these beach sands were related to the presence of oil.

For the water samples, the temporal variability and general lack of coherent patterns among our measured variables precluded identification of community patterns directly related to oiled beaches. However, in the OB June sample, when oil was visibly present in the water, the community composition was distinct from all other samples (Figure 5). The community categorization analysis suggests these differences were driven strongly by OTUs in the resident community categorized as belonging to the genera *Alcanivorax* and *Gracilimonas* (Figure 7). Little is known about the genus *Gracilimonas*, so its association with oiled beaches in our study may not indicate an ability to degrade oil hydrocarbons. *Alcanivorax* , a known oil hydrocarbon degrader in the marine environment [15,16], the only genus that distinguished both freshly-oiled water and sand samples in our study, and a prominent taxon associated with oiled sands at *Pensacola* Beach [6], appears to be a major player in the degradation of oil hydrocarbons along Gulf Coast beaches.

## Supporting Information

Figure S1 **Dendrogram illustrating the bacterial community composition relationships among prevalence groups.** An average-group linkage dendrogram is illustrated for the core (≥75% of samples), resident (25-75% of samples), and transient (<25% of samples) OTU communities for exposed sand (left) and water (right) samples. See methods for category breakdown details. The mean OTU composition is represented for beaches with multiple sequenced samples from the same date. Sample features are indicated with colored boxes according to the key. For example, the first rectangle next to the beach name represents samples collected from either west of Mobile Bay (blue) or east of Mobile Bay (yellow).
(TIF)

Table S1 Beach conditions during sample collection.
(PDF)

Table S2 **List of taxonomic groups contributing >0.5% of the total sequence reads recovered from an environment.**
(PDF)
