# Correction: Shifts in the Microbial Community Composition of Gulf Coast Beaches Following Beach Oiling

**DOI:** 10.1371/journal.pone.0094671

**Published:** 2014-04-02

**Authors:** The PLOS ONE Staff

Figure S1 is a duplicate of Table S2. This error occurred while the article was being prepared by production for publication. The correct Figure S1 can be viewed below.

## Supporting Information

Figure S1 **Dendrogram illustrating the bacterial community composition relationships among prevalence groups.** An average-group linkage dendrogram is illustrated for the core (≥75% of samples), resident (25-75% of samples), and transient (<25% of samples) OTU communities for exposed sand (left) and water (right) samples. See methods for category breakdown details. The mean OTU composition is represented for beaches with multiple sequenced samples from the same date. Sample features are indicated with colored boxes according to the key. For example, the first rectangle next to the beach name represents samples collected from either west of Mobile Bay (blue) or east of Mobile Bay (yellow).

## References

[1] Newton RJ, Huse SM, Morrison HG, Peake CS, Sogin ML, et al (2013) Shifts in the Microbial Community Composition of Gulf Coast Beaches Following Beach Oiling. PLoS ONE 8(9): e74265 doi:10.1371/journal.pone.0074265 24040219 10.1371/journal.pone.0074265 PMC3769389

