# Supplementary material for: Shifts in the Microbial Community Composition of Gulf Coast Beaches Following Beach Oiling
Source: PLoS One. 2013 Sep 10;8(9):e74265. doi: 10.1371/journal.pone.0074265 (PMC3769389; doi:10.1371/journal.pone.0074265)
Supplement: Table S1 — Beach conditions during sample collection. (PDF) [file pone.0074265.s002.pdf]

Table S1. Beach Conditions during Sample Collection

Beach[1]	Sample Date	Time (local)	Wind Speed (m/s)[2]	Water Temp (°C)[2]	Precipitation (48 hour, inches)[2]	Weather Notes	Hold Time (hours)[3]
St George Island	13-Jun-10	16:45	2	29.9	0	Sunny & hot & calm	25
Henderson	15-Jun-10	10:45	1.5	30.4	0	Sunny & hot & calm	7
Fort Pickens	14-Jun-10	11:30	1.13	29.9	0	Sunny & hot & calm	22
Orange Beach	15-Jun-10	11:15	2.7	31.4	0	Sunny & hot & calm	28
St. Andrews	15-Jun-10	13:45	3.08	29.6	0	Sunny & hot & calm	19
Gulfport East	14-Jun-10	19:15	3.08	N/A	0	Sunny & hot & calm	22
Bay St. Louis	16-Jun-10	8:30	3.8	N/A	0	Sunny & hot & calm	7
St George Island	N/A						
Henderson	9-Aug-10	10:30	3.6	30.9	0.03	Sunny & calm	7
Fort Pickens	8-Aug-10	18:00	2.4	32.3	1.77	Large Rain storm just before sampling, light rain during sampling	18
Orange Beach	8-Aug-10	16:00	1.5	31.2	0.62	Large Rain storm just before sampling, light rain during sampling	20
St. Andrews	10-Aug-10	11:00	3.08	30.2	3.74	Large Rain storm just before sampling	6
Gulfport East	9-Aug-10	17:00	3.4	N/A	0	Sunny & calm	24
Bay St. Louis	9-Aug-10	14:00	3.7	30.4	0	Sunny & calm	27
St George Island	20-Sep-10	16:30	2.5	30.1	0	Sunny & high surf	24
Henderson	21-Sep-10	14:30	2.4	29.8	0	Thick algal bloom in water & high surf	2
Fort Pickens	22-Sep-10	12:00	1.4	29.8	0	Thick algal bloom in water & high surf	21
Orange Beach	22-Sep-10	16:00	7.4	30.7	0	High Surf	17
St. Andrews	20-Sep-10	15:30	1.56	30.3	0	Sunny & calm	17
Gulfport East	19-Sep-10	16:00	2.6	N/A	0	Sunny & calm	17
Bay St. Louis	19-Sep-10	14:30	2.2	29.1	0	Sunny & calm	16
St George Island	15-Nov-10	14:45	6.4	21	0	Windy & high surf	24
Henderson	16-Nov-10	16:00	10.7	20.2	3.11	Very high surf, water 10-50 m further inland, sampled post heavy rain	2
Fort Pickens	17-Nov-10	9:30	4.7	19.3	0.9	Windy & high surf	21
Orange Beach	18-Nov-10	8:45	5.3	22.3	0	Windy & high surf	3
St. Andrews	17-Nov-10	8:30	3.6	18.9	0.08	Sunny & calm	8
Gulfport East	16-Nov-10	8:00	2.6	N/A	0.98	Rain storm prior to sampling	4
Bay St. Louis	15-Nov-10	16:15	1.6	19	0.21	Rain storm prior to sampling	20

[1] Sample coordinates for each beach site are listed below

	Site 1 (lat, lon)	Site 2 (lat, lon)	Site 3 (lat, lon)
St George Island	29.6891 -84.7836	29.6894 -84.7830	29.6894, -84.7825
Henderson	30.3831 -86.4445	30.3830 -86.4428	30.3831, -86.4428
Fort Pickens	30.3170 -87.2573	30.3169 -87.2595	30.3168 -87.2624
Orange Beach	30.2692 -87.5805	30.2689 -87.5819	30.2689, -87.5831
St. Andrews	30.3428 -88.7071	30.3428 -88.7079	30.3428 -88.7088
Gulfport East	30.3816 -89.0260	30.3813 -89.0272	30.3812 -89.0282
Bay St. Louis	30.2976 -89.3366	30.2979 -89.3361	30.2989, -89.3353

[2] Data collected from NOAA coastal monitoring program, National Oceanographic Data Center at locations nearest to sampling site

[3] Hold time on ice before samples were frozen
