# Supplementary material for: Shifts in the Microbial Community Composition of Gulf Coast Beaches Following Beach Oiling
Source: PLoS One. 2013 Sep 10;8(9):e74265. doi: 10.1371/journal.pone.0074265 (PMC3769389; doi:10.1371/journal.pone.0074265)
Supplement: Table S2 — List of taxonomic groups contributing >0.5% of the total sequence reads recovered from an environment. (PDF) [file pone.0074265.s003.pdf]

Table S2. List of taxonomic groups contributing >0.5% of the total sequence reads recovered from an environment

Exposed Sand

Taxonomy	% Total Reads
Bacteria; Proteobacteria; Gammaproteobacteria; Xanthomonadales; Sinobacteraceae; genus NA	11.63%
Bacteria; Bacteroidetes; Flavobacteria; Flavobacteriales; Flavobacteriaceae; Zeaxanthinibacter	7.51%
Bacteria; Bacteroidetes; Sphingobacteria; Sphingobacteriales; Saprospiraceae; Haliscomenobacter	5.30%
Bacteria; Proteobacteria; Gammaproteobacteria; order NA; family NA; genus NA	3.88%
Bacteria; Actinobacteria; Actinobacteria, Acidimicrobiales; family NA; genus NA	2.94%
Bacteria; Proteobacteria; Deltaproteobacteria; order NA; family NA; genus NA	1.98%
Bacteria; Bacteroidetes; Flavobacteria; Flavobacteriales; Flavobacteriaceae; genus NA	1.96%
Bacteria; Chloroflexi; Caldilineae; Caldilineales; family NA; genus NA	1.74%
Bacteria; Proteobacteria; Alphaproteobacteria; Sphingomonadales; Erythrobacteraceae; Erythrobacter	1.73%
Bacteria; Bacteroidetes; Sphingobacteria; Sphingobacteriales; Rhodothermaceae; genus NA	1.58%
Bacteria; Bacteroidetes; Sphingobacteria; Sphingobacteriales; Saprospiraceae; genus NA	1.56%
Bacteria; Proteobacteria; Deltaproteobacteria; Myxococcales; family NA; genus NA	1.54%
Bacteria; Planctomycetes; Planctomycetacia; Planctomycetales; Planctomycetaceae; genus NA	1.44%
Bacteria; Bacteroidetes; Sphingobacteria; Sphingobacteriales; Chitinophagaceae; genus NA	1.31%
Bacteria; Bacteroidetes; Flavobacteria; Flavobacteriales; Flavobacteriaceae; Winogradskyella	1.27%
Bacteria; Proteobacteria; Gammaproteobacteria; Alteromonadales; Alteromonadaceae; Alteromonas	1.12%
Bacteria; Planctomycetes; Planctomycetacia; Planctomycetales; Planctomycetaceae; Blastopirellula	1.11%
Bacteria; Bacteroidetes; Sphingobacteria; Sphingobacteriales; family NA; genus NA	1.11%
Bacteria; Planctomycetes; Planctomycetacia; Planctomycetales; Planctomycetaceae; Rhodopirellula	1.01%
Bacteria; Proteobacteria; Gammaproteobacteria; Alteromonadales; Alteromonadaceae; Marinobacter	1.01%
Bacteria; Acidobacteria; Acidobacteria Gp22; order NA; family NA; genus NA	0.86%
Bacteria; Bacteroidetes; Sphingobacteria; Sphingobacteriales; Cytophagaceae; Pontibacter	0.84%
Bacteria; Proteobacteria; Gammaproteobacteria; Chromatiales; Chromatiaceae; Nitrosococcus	0.83%
Bacteria; Bacteroidetes; Flavobacteria; Flavobacteriales; Flavobacteriaceae; Robiginitalea	0.82%
Bacteria; Proteobacteria; Alphaproteobacteria; Rhodobacterales; Rhodobacteraceae; genus NA	0.79%
Bacteria; Bacteroidetes; Sphingobacteria; Sphingobacteriales; Flammeovirgaceae; genus NA	0.79%
Bacteria; WS3; class NA; order NA; family NA; genus NA	0.78%
Bacteria; Proteobacteria; Deltaproteobacteria; Desulfobacterales; Desulfobacteraceae; genus NA	0.74%
Bacteria; Acidobacteria; Holophagae; order NA; family NA; genus NA	0.73%
Bacteria; Chloroflexi; Anaerolineae; Anaerolineales; Anaerolinaceae; genus NA	0.72%
Bacteria; Deferribacteres; Deferribacteres; Deferribacterales; Unassigned; Caldithrix	0.70%
Bacteria; Bacteroidetes; Sphingobacteria; Sphingobacteriales; Saprospiraceae; Lewinella	0.67%
Bacteria; Proteobacteria; Gammaproteobacteria; Alteromonadales; Alteromonadaceae; Salinimonas	0.66%
Bacteria; Proteobacteria; Gammaproteobacteria; Alteromonadales; Alteromonadaceae; genus NA	0.62%
Bacteria; Gemmatimonadetes; Gemmatimonadetes; order NA; family NA; genus NA	0.62%
Bacteria; Bacteroidetes; Sphingobacteria; Sphingobacteriales; Cytophagaceae; Flexibacter	0.61%
Bacteria; Actinobacteria; Actinobacteria, Acidimicrobiales; Iamiaceae; Iamia	0.60%
Bacteria; Verrucomicrobia; Verrucomicrobiae; Verrucomicrobiales; Verrucomicrobiaceae; genus NA	0.60%
Bacteria; Acidobacteria; Acidobacteria; Acidobacteriales; Acidobacteriaceae; Chloroacidobacterium	0.59%
Bacteria; Proteobacteria; Alphaproteobacteria; Rhodobacterales; Rhodobacteraceae; Palleronia	0.58%

Submerged Sand

Taxonomy	% Total Reads
Bacteria; Proteobacteria; Gammaproteobacteria; Xanthomonadales; Sinobacteraceae; genus NA	10.83%
Bacteria; Bacteroidetes; Flavobacteria; Flavobacteriales; Flavobacteriaceae; Zeaxanthinibacter	6.52%
Bacteria; Bacteroidetes; Sphingobacteria; Sphingobacteriales; Saprospiraceae; Haliscomenobacter	5.86%
Bacteria; Proteobacteria; Gammaproteobacteria; order NA; family NA; genus NA	3.93%
Bacteria; Actinobacteria; Actinobacteria, Acidimicrobiales; family NA; genus NA	3.67%

Bacteria; Bacteroidetes; Flavobacteria; Flavobacteriales; Flavobacteriaceae; genus NA	2.58%
Bacteria; Proteobacteria; Deltaproteobacteria; order NA; family NA; genus NA	2.26%
Bacteria; Chloroflexi; Caldilineae; Caldilineales; family NA; genus NA	2.08%
Bacteria; Planctomycetes; Planctomycetacia; Planctomycetales; Planctomycetaceae; Blastopirellula	1.88%
Bacteria; Proteobacteria; Deltaproteobacteria; Myxococcales; family NA; genus NA	1.69%
Bacteria; Bacteroidetes; Flavobacteria; Flavobacteriales; Flavobacteriaceae; Winogradskyella	1.54%
Bacteria; Planctomycetes; Planctomycetacia; Planctomycetales; Planctomycetaceae; genus NA	1.52%
Bacteria; Bacteroidetes; Sphingobacteria; Sphingobacteriales; Saprospiraceae; genus NA	1.50%
Bacteria; Bacteroidetes; Sphingobacteria; Sphingobacteriales; Chitinophagaceae; genus NA	1.47%
Bacteria; Bacteroidetes; Sphingobacteria; Sphingobacteriales; Rhodothermaceae; genus NA	1.30%
Bacteria; Bacteroidetes; Flavobacteria; Flavobacteriales; Flavobacteriaceae; Robiginitalea	1.30%
Bacteria; Bacteroidetes; Sphingobacteria; Sphingobacteriales; family NA; genus NA	1.20%
Bacteria; Planctomycetes; Planctomycetacia; Planctomycetales; Planctomycetaceae; Rhodopirellula	1.18%
Bacteria; Chloroflexi; Anaerolineae; Anaerolineales; Anaerolinaceae; genus NA	1.16%
Bacteria; Verrucomicrobia; Verrucomicrobiae; Verrucomicrobiales; Verrucomicrobiaceae; genus NA	1.01%
Bacteria; Proteobacteria; Alphaproteobacteria; Sphingomonadales; Erythrobacteraceae; Erythrobacter	0.93%
Bacteria; Bacteroidetes; Sphingobacteria; Sphingobacteriales; Flammeovirgaceae; genus NA	0.92%
Bacteria; Proteobacteria; Deltaproteobacteria; Desulfobacterales; Desulfobacteraceae; genus NA	0.90%
Bacteria; WS3; class NA; order NA; family NA; genus NA	0.87%
Bacteria; Bacteroidetes; Sphingobacteria; Sphingobacteriales; Saprospiraceae; Lewinella	0.86%
Bacteria; Proteobacteria; Alphaproteobacteria; Rhodobacterales; Rhodobacteraceae; genus NA	0.85%
Bacteria; Acidobacteria; Acidobacteria Gp22; order NA; family NA; genus NA	0.81%
Bacteria; Acidobacteria; Holophagae; order NA; family NA; genus NA	0.77%
Bacteria; Deferribacteres; Deferribacteres; Deferribacterales; Unassigned; Caldithrix	0.69%
Bacteria; Proteobacteria; Gammaproteobacteria; Chromatiales; Chromatiaceae; Nitrosococcus	0.69%
Bacteria; Actinobacteria; Actinobacteria, Acidimicrobiales; Iamiaceae; Iamia	0.63%
Bacteria; Acidobacteria; Acidobacteria; Acidobacteriales; Acidobacteriaceae; Chloroacidobacterium	0.60%
Bacteria; Gemmatimonadetes; Gemmatimonadetes; order NA; family NA; genus NA	0.60%
Bacteria; Lentisphaerae; Lentisphaeria; order NA; family NA; genus NA	0.59%
Bacteria; Proteobacteria; Gammaproteobacteria; Acidithiobacillales; Acidithiobacillaceae; Acidithiobacillus	0.58%
Bacteria; Proteobacteria; Gammaproteobacteria; Alteromonadales; Alteromonadaceae; genus NA	0.54%
Bacteria; Chlorobia; Chlorobia; Chlorobiales; family NA; genus NA	0.53%
Bacteria; phylum NA; class NA; order NA; family NA; genus NA	0.53%
Bacteria; Acidobacteria; Acidobacteria; Acidobacteriales; Acidobacteriaceae; genus NA	0.52%

Water

Taxonomy	% Total Reads
Bacteria; Cyanobacteria; Subsection I; Unassigned; Synechococcus	17.64%
Bacteria; Proteobacteria; Alphaproteobacteria; Rickettsiales; SAR11; Pelagibacter	11.20%
Bacteria; Proteobacteria; Alphaproteobacteria; Rhodobacterales; Rhodobacteraceae; genus NA	5.47%
Bacteria; Actinobacteria; Actinobacteria, Acidimicrobiales; family NA; genus NA	4.47%
Bacteria; Bacteroidetes; Flavobacteria; Flavobacteriales; Flavobacteriaceae; genus NA	3.95%
Bacteria; Proteobacteria; Gammaproteobacteria; Oceanospirillales; SAR86; genus NA	3.83%
Bacteria; Proteobacteria; Alphaproteobacteria; Rhodospirillales; Rhodospirillaceae; genus NA	2.23%
Bacteria; Proteobacteria; Alphaproteobacteria; Rickettsiales; SAR11; genus NA	2.05%
Bacteria; Bacteroidetes; Flavobacteria; Flavobacteriales; Cryomorphaceae; Owenweeksia	1.86%
Bacteria; Proteobacteria; Alphaproteobacteria; Rickettsiales; SAR116; genus NA	1.73%
Bacteria; Proteobacteria; Gammaproteobacteria; Alteromonadales; Alteromonadaceae; Hailea	1.61%
Bacteria; Bacteroidetes; Sphingobacteria; Sphingobacteriales; Chitinophagaceae; Gracilimonas	1.56%
Bacteria; Bacteroidetes; Sphingobacteria; Sphingobacteriales; Saprospiraceae; Haliscomenobacter	1.47%
Bacteria; Cyanobacteria; Subsection I; family NA; genus NA	1.12%
Bacteria; Proteobacteria; Gammaproteobacteria; order NA; family NA; genus NA	1.11%
Bacteria; Proteobacteria; Deltaproteobacteria; SAR324; family NA; genus NA	0.97%
Bacteria; Proteobacteria; Gammaproteobacteria; Pseudomonadales; Moraxellaceae; Acinetobacter	0.97%
Bacteria; Proteobacteria; Gammaproteobacteria; Xanthomonadales; Sinobacteraceae; genus NA	0.81%
Bacteria; Planctomycetes; Planctomycetacia; Planctomycetales; Planctomycetaceae; genus NA	0.78%

Bacteria; Proteobacteria; Betaproteobacteria; Burkholderiales; Alcaligenaceae; genus NA	0.73%
Bacteria; Actinobacteria; Actinobacteria; Actinomycetales; Microbacteriaceae; Aquiluna	0.72%
Bacteria; Proteobacteria; Gammaproteobacteria; Alteromonadales; Alteromonadaceae; genus NA	0.72%
Bacteria; Bacteroidetes; Sphingobacteria; Sphingobacteriales; family NA; genus NA	0.68%
Bacteria; Proteobacteria; Gammaproteobacteria; Oceanospirillales; family NA; genus NA	0.67%
Bacteria; Bacteroidetes; Flavobacteria; Flavobacteriales; Flavobacteriaceae; Croceitalea	0.66%
Bacteria; Actinobacteria; Actinobacteria; Acidimicrobiales; Acidimicrobiaceae; genus NA	0.66%
Bacteria; Bacteroidetes; Flavobacteria; Flavobacteriales; family NA; genus NA	0.63%
Bacteria; Proteobacteria; Deltaproteobacteria; Myxococcales; family NA; genus NA	0.62%
Bacteria; Bacteroidetes; Sphingobacteria; Sphingobacteriales; Chitinophagaceae; Baineola	0.58%
Bacteria; Actinobacteria; Actinobacteria; Actinomycetales; family NA; genus NA	0.56%
Bacteria; Bacteroidetes; Sphingobacteria; Sphingobacteriales; Chitinophagaceae; genus NA	0.54%
Bacteria; Bacteroidetes; Sphingobacteria; Sphingobacteriales; Saprospiraceae; genus NA	0.51%
