# Supplementary figures and images for: Correction: Shifts in the Microbial Community Composition of Gulf Coast Beaches Following Beach Oiling
Source: PLoS One. 2014 Apr 2;9(4):e94671. doi: 10.1371/journal.pone.0094671 (PMC3973571; doi:10.1371/journal.pone.0094671)

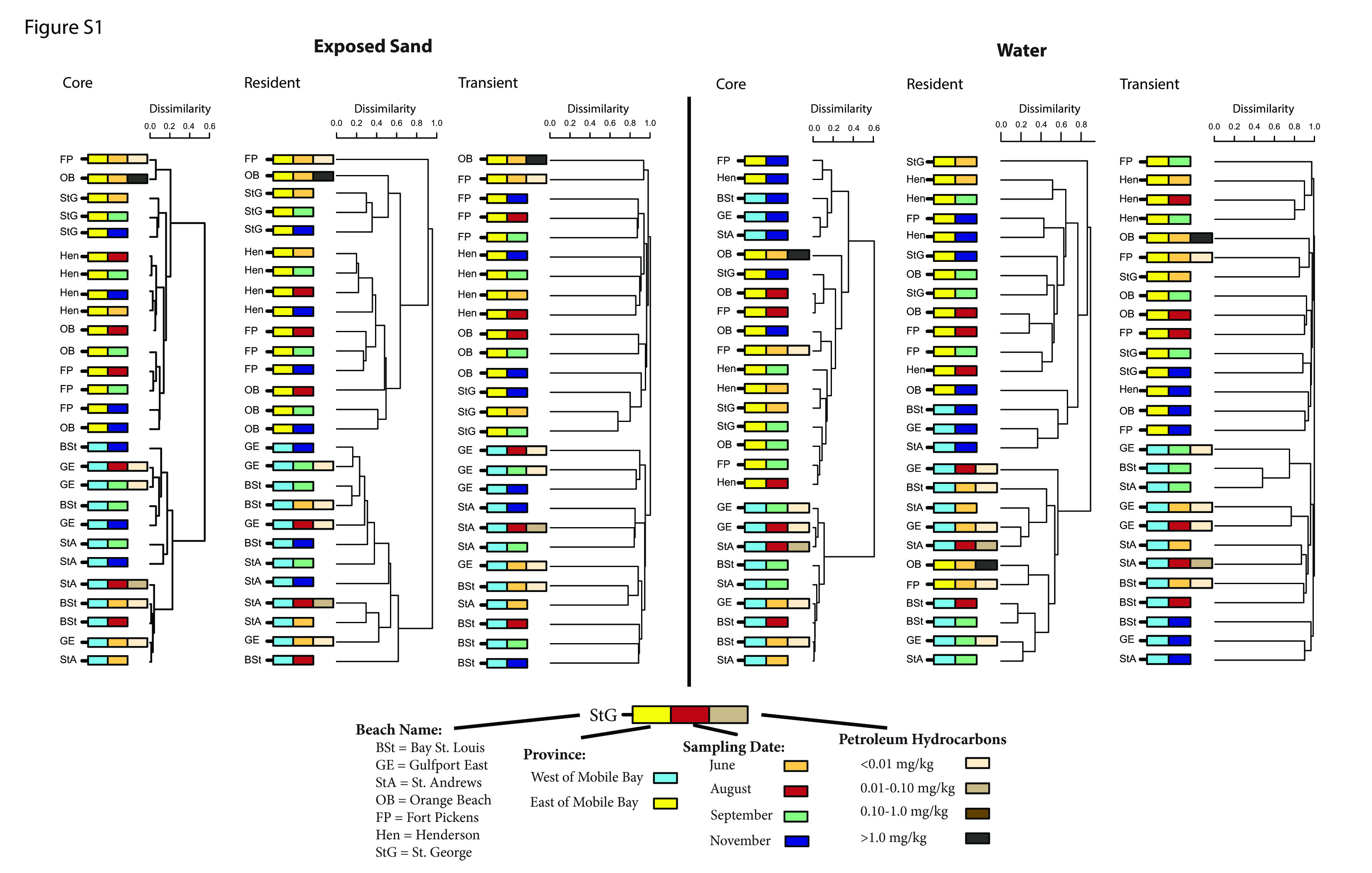

Supplement: Figure S1 — Dendrogram illustrating the bacterial community composition relationships among prevalence groups. An average-group linkage dendrogram is illustrated for the core (≥75% of samples), resident (25-75% of samples), and transient (<25% of samples) OTU communities for exposed sand (left) and water (right) samples. See methods for category breakdown details. The mean OTU composition is represented for beaches with multiple sequenced samples from the same date. Sample features are indicated with colored boxes according to the key. For example, the first rectangle next to the beach name represents samples collected from either west of Mobile Bay (blue) or east of Mobile Bay (yellow). [file pone.0094671.s001.tif]
